# Profound *N*-glycan remodelling accompanies MHC-II immunopeptide presentation

**DOI:** 10.3389/fimmu.2023.1258518

**Published:** 2023-11-08

**Authors:** Hayley Goodson, Rebeca Kawahara, Sayantani Chatterjee, Gabriel Goncalves, Joshua Fehring, Anthony W. Purcell, Nathan P. Croft, Morten Thaysen-Andersen

**Affiliations:** ^1^ School of Natural Sciences, Macquarie University, Sydney, NSW, Australia; ^2^ Institute for Glyco-core Research (iGCORE), Nagoya University, Nagoya, Japan; ^3^ Department of Biochemistry & Cell Biology, Boston University Chobanian & Avedisian School of Medicine, Boston, MA, United States; ^4^ Department of Biochemistry and Molecular Biology, Monash Biomedicine Discovery Institute, Monash University, Clayton, VIC, Australia

**Keywords:** MHC-II, immunopeptides, *N*-glycans, glycan remodelling, glycomics, glycoproteomics

## Abstract

Immunopeptidomics, the study of peptide antigens presented on the cell surface by the major histocompatibility complex (MHC), offers insights into how our immune system recognises self/non-self in health and disease. We recently discovered that hyper-processed (remodelled) *N*-glycans are dominant features decorating viral spike immunopeptides presented via MHC-class II (MHC-II) molecules by dendritic cells pulsed with SARS-CoV-2 spike protein, but it remains unknown if endogenous immunopeptides also undergo *N*-glycan remodelling. Taking a multi-omics approach, we here interrogate published MHC-II immunopeptidomics datasets of cultured monocyte-like (THP-1) and breast cancer-derived (MDA-MB-231) cell lines for overlooked *N*-glycosylated peptide antigens, which we compare to their source proteins in the cellular glycoproteome using proteomics and *N*-glycomics data from matching cell lines. Hyper-processed chitobiose core and paucimannosidic *N-*glycans alongside under-processed oligomannosidic *N*-glycans were found to prevalently modify MHC-II-bound immunopeptides isolated from both THP-1 and MDA-MB-231, while complex/hybrid-type *N*-glycans were (near-)absent in the immunopeptidome as supported further by new *N*-glycomics data generated from isolated MHC-II-bound peptides derived from MDA-MB-231 cells. Contrastingly, the cellular proteomics and *N*-glycomics data from both cell lines revealed conventional *N*-glycosylation rich in complex/hybrid-type *N*-glycans, which, together with the identification of key lysosomal glycosidases, suggest that MHC-II peptide antigen processing is accompanied by extensive *N*-glycan trimming. *N*-glycan remodelling appeared particularly dramatic for cell surface-located glycoproteins while less remodelling was observed for lysosomal-resident glycoproteins. Collectively, our findings indicate that both under- and hyper-processed *N*-glycans are prevalent features of endogenous MHC-II immunopeptides, an observation that demands further investigation to enable a better molecular-level understanding of immune surveillance.

## Introduction

Immune surveillance is a critical mechanism employed by our immune system to monitor for and protect against foreign agents posing health threats. The major histocompatibility complex (MHC) class I (MHC-I) and class II (MHC-II) molecules present repertoires of immunopeptides on the surface of cells, the immunopeptidome, by which T-cells can recognise abnormal or foreign peptide antigens, such as those of pathogen origin (1). The MHC molecules are encoded by highly polymorphic genes, giving rise to a wide spectrum of MHC variants (in humans referred to as human leukocyte antigen [HLA] allotypes) each displaying different peptide binding specificities leading to highly diverse populations of immunopeptides.

In addition to the inherent genetic variability of the MHC molecules, non-coded post-translational modifications (PTMs) of proteins greatly expand the molecular and functional diversity of the immunopeptidome (2, 3). PTMs are known to impact the chemical structure, presentation and, ultimately, the function of immunopeptides thereby playing key roles in shaping the immune response to pathogenic and other health-detrimental insults (4, 5).

Facilitated by advances in mass spectrometry (MS), the immunopeptidome can now be comprehensively profiled with high sensitivity and precision using established liquid chromatography-tandem MS (LC-MS/MS) methods (6). However, the existing LC-MS/MS-based immunopeptidomics protocols for sample preparation and data acquisition/analysis are still focused largely on identifying unmodified immunopeptides, leaving, in most cases, their modified counterparts unrecognised.

Asparagine- (Asn-, *N-*) linked glycosylation is a common and complex PTM known to impact the structural and functional diversity of the cellular proteome (7, 8). While to date there have only been relatively few studies reporting on *N-*glycosylation of MHC-bound immunopeptides, building evidence supports that in particular the MHC-II immunopeptidome contains a significant proportion of peptide antigens bearing *N*-glycans. In an early report, remnants of the *N*-glycan trimannosylchitobiose core were identified from naturally processed MHC-II immunopeptides bound to HLA-DR alleles (9). Several years later, HLA-DR-bound MHC-II immunopeptides were again reported to carry unusually short *N*-glycans including a paucimannose-type *N*-glycan with an intact trimannosylchitobiose core (Man_3_GlcNA_2_Fuc_1_) (10). In further support, Malaker and co-workers explored the *N-*glycosylation of MHC-II-bound immunopeptides presented by cultured melanoma and matched EBV-transformed B lymphoblastoid cells and found that a variety of *N*-glycan structures including paucimannosidic-, oligomannosidic- and complex/hybrid-type *N*-glycans decorate MHC-II-bound immunopeptides presented on those cell lines (11). Finally, enabled by new data analysis software tailored for glycopeptide identification, Nesvizhskii and colleagues recently reported on the *N*-glycosylation of immunopeptides from a variety of cell lines by re-interpreting previously published immunopeptidomics data (12). These efforts led to a valuable library (the ‘HLA-Glyco database’) containing over 3,400 *N*-glycosylated MHC-II-bound immunopeptides across 1,049 glycosylation sites providing robust evidence to support that *N*-glycosylation is wide-spread within the MHC-II immunopeptidome.

Notably, glycosylation of immunopeptides has also been shown to be important for the specificity of some human MHC-II-restricted T-cells (13–15). Forming a foundation of this present study, we have recently found that dendritic cells (DCs) phagocytose and process the highly *N-*glycosylated SARS-CoV-2 spike protein and prevalently present MHC-II-bound immunopeptides carrying both under- and hyper-processed (remodelled) spike *N*-glycans on their surfaces including oligomannosidic (Man_5-9_GlcNAc_2_) and paucimannosidic (Man_1-3_GlcNAc_2_Fuc_0-1_) *N*-glycans for T-cell recognition, starkly contrasting the glycophenotype of the native spike protein that was shown to be rich in elongated complex-type *N*-glycans (16). While the unusual glycosylation features of these immunopeptides provide clues regarding how glycans decorating exogenous viral glycoproteins are perceived and altered by our immune system, it remains unknown if endogenous (human) immunopeptides also carry remodelled *N*-glycans that differ from their native cellular glycoproteins, details that are important to enable a better understanding of the glycobiology underpinning immunopeptide presentation and recognition.

Taking a multi-omics approach, we here explore the *N-*glycosylation of endogenous immunopeptides by interrogating published MHC-II immunopeptidomics datasets and matching *N*-glycomics and proteomics data of cell lysates from cultured monocyte-like (THP-1) and breast cancer-derived (MDA-MB-231) cells. We find evidence to support the notion that both under-processed and extensively remodelled *N*-glycans prevalently modify endogenous MHC-II immunopeptides.

## Methods

The experimental approach has been summarised in **Supplementary Figure S1A**.

### Data origin and selection criteria

Publicly available MHC-II immunopeptidomics LC-MS/MS datasets with matching LC-MS/MS-based proteomics and PGC-LC-MS/MS-based *N*-glycomics datasets of the cellular (lysate) fraction were identified by, firstly, searching the PRIDE repository against the keywords “immunopeptidomics” and “MHC-II” (17), and, secondly, by searching the literature for matching high-quality proteomics and glycomics data of the same biological systems, **Supplementary Figure S1B**. Glycopeptide enrichment was neither performed for the selected immunopeptidome nor for the used proteome data to avoid any analytical bias that glycopeptide enrichment may cause. The retrieved immunopeptidomics and proteomics datasets were manually inspected and only studies with high-quality LC-MS/MS data (robust chromatography, high fragmentation efficiency and with acceptable spectral signal-to-noise) generated using HCD-MS/MS fragmentation acquired on high-resolution mass spectrometers were selected. HCD-MS/MS spectra of glycopeptides are namely known to contain 1) diagnostic oxonium ions (e.g. *m/z* 204.0867 for HexNAc) specifically reporting on the presence of glycopeptides and other fragment ions including 2) peptide b-/y-ions, 3) glycopeptide Y-ions, and 4) glycan B-ions, required for confident glycopeptide identification. MHC-II immunopeptidomics datasets from a monocyte-like cell line [THP-1, i.e. PXD015646 and PXD005169 (18, 19)] and a triple negative breast cancer cell line [MDA-MB-231, i.e. PXD023044, PXD023038 and MSV000083727 (20, 21)] each comprising technical replicates generated across different culture conditions met these criteria. For the THP-1 immunopeptidomics data (18), all experimental conditions were used to validate that the identified glycosylated and non-glycosylated immunopeptides conformed to the expected MHC-II characteristics, while only the phorbol myristate acetate (PMA)-treated condition was used for the glycoprofiling to enable a fair comparison between the glycosylation of immunopeptides and the cellular glycoproteome. Raw data (immunopeptidomics, *N*-glycomics, proteomics) for the two investigated systems meeting the above conditions and of an acceptable data quality were downloaded and searched as described below.

### Interrogation of published immunopeptidomics and cellular proteomics data

All immunopeptidomics and proteomics LC-MS/MS raw data were searched for *N*-glycopeptides using Byonic v3.9.4 (Protein Metrics) operated using identical search settings with the exception of the enzyme specificity (**Supplementary Figure S1C**). The proteomics datasets were searched against fully specific tryptic peptides with up to two missed cleavages permitted while the immunopeptidomics datasets were searched using non-specific R/K digestion settings with up to two missed cleavages allowed per peptide to speed up the search by limiting the peptide search space without biasing towards tryptic cleavage patterns (see below). The generated HCD-MS/MS spectra were searched using the following search settings: precursor/product ions were permitted to deviate up to 10/20 ppm from the expected (theoretical) values, up to one *N*-glycan per peptide was allowed as a variable ‘common’ modification, and up to one Met oxidation (+15.994 Da) or one Asn deamidation (+0.984 Da) was permitted per peptide as a variable ‘common’ modification. Monoisotopic correction (error check equals +/- floor, mass in Da/4,000) was used. For the glycan search space, a glycomics-guided *N-*glycan database containing all *N*-glycans identified from the available *N*-glycomics data of THP-1 and MDA-MB-231 lysates (see below) was generated for each of the two biological systems. Importantly, ultra-truncated *N*-glycans known to go undetected in our glycomics approach were manually added to the *N*-glycan search space in both biological systems. All data were searched against the entire human proteome (UniProt ID: 9606) using a decoy database and a list of common contaminants available in Byonic to enable FDR control.

From the Byonic search output, all glycopeptide candidates were firstly filtered to PEP 2D < 0.01 and any glycopeptides from the reverse and contaminant proteins manually deleted. To improve confidence further, the immunopeptidomics datasets were additionally filtered by peptide length, only including the expected MHC-II peptide length (12-25 amino acid residues). Finally, the filtered glycopeptides were assessed for nested sets and the biosynthetic relatedness of the observed glycoforms. For the proteomics datasets, the Byonic search output was further filtered by excluding the bottom 10% least confident glycoPSMs in both the MDA-MB-231 and THP-1 datasets for increased confidence in the reported tryptic glycopeptides. The quantitation of observed glycopeptides in both the immunopeptidomics and cellular proteomics data was based on spectral counting of the glycopeptide-to-spectrum matches (glycoPSMs). To determine glycopeptide prevalence in the immunopeptidomics raw data, the MS/MS spectra and glycosylated MS/MS spectra in the LC-MS/MS files were counted using in-house software that searches for the presence of oxonium ions (*m/z* 204.0867 ± 10 ppm with an intensity threshold of 10% of maximum intensity, and a minimum of five fragment ions per spectrum) as diagnostic reporters of glycopeptides.

### Reinterrogation of published cellular *N*-glycomics data

The LC-MS/MS-based *N*-glycomics raw data files of MDA-MB-231 and THP-1 were browsed and inspected using ESI-Compass Data Analysis 4.0 software v1.1 (Bruker Daltonics). *N*-glycans were identified based on their monoisotopic precursor mass, absolute and relative PGC-LC retention time and MS/MS fragmentation pattern as described (22, 23). The relative abundances of the individual *N*-glycans were determined based on area-under-the-curve measurements from extracted ion chromatograms of the monoisotopic precursor ions using Skyline v22.2 and QuantAnalysis v2.1 (Bruker Daltonics). Glycans were depicted using the latest symbol nomenclature for graphical representation of glycans (24). Glycan depiction and spectral annotation were assisted by the GlycoWorkBench v2.1 software.

### Generation of new *N*-glycomics data of isolated MDA-MB-231 immunopeptides

Isolated MHC-II-bound immunopeptides from the MDA-MB-231 cell line were available from our previous study (20). These immunopeptides were subjected to *N*-glycome profiling using an established glycomics method with minor modifications (25). In short, the MHC-II immunopeptides were in-solution de-*N*-glycosylated using 20 U recombinant peptide:*N*-glycosidase F (PNGase F, *Elizabethkingia miricola*, Promega) in 20 µl 10 mM ammonium bicarbonate for 14 h at 37°C. The detached *N-*glycans and the formerly *N*-glycosylated immunopeptides were then applied to primed Oligo R3 reversed phase solid phase extraction (SPE) micro-columns. The *N*-glycans, which were found in the flowthrough/wash fractions, were reduced to alditols using sodium borohydride and desalted using custom-made porous graphitised carbon (PGC) SPE micro-columns. The *N*-glycans were detected using an established PGC-LC–MS/MS method (26). In short, the *N*-glycans were injected on a heated (50°C) Hypercarb KAPPA PGC HPLC column (1 mm internal diameter, 30 mm length, 3 μm particle size, 250 Å pore size, Thermo Fisher Scientific). The *N*-glycans were separated over a 60 min multi-step gradient of solvent B containing 70% ACN in 10 mM aqueous ammonium bicarbonate (solvent A). The gradient started at 0% B (0-3 min), increased to 14% B (3-4 min), 40% B (4-40 min), 56% B (40-48 min), 100% B (48-50 min), kept at 100% B (50-54 min), dropped to 0% B (54-56 min) and then stayed at 0% B (56-60 min). A constant flow rate of 20 µL/min was maintained with a post-column make-up flow supplying 70% ACN delivered by a Dionex Ultimate-3000 HPLC system (Thermo Fisher Scientific). The separated *N*-glycans were ionised using electrospray ionisation and detected in negative ion polarity mode using a linear trap quadrupole Velos Pro ion trap mass spectrometer (Thermo Fisher Scientific) with a full scan acquisition range of *m/z* 660–2,000 and a source voltage of +2.75 kV. The automatic gain control for the MS1 scans was set to 3 × 10^4^ with a maximum accumulation time of 100 ms. For MS/MS, the automatic gain control was 2 × 10^4^ and the maximum accumulation time was 300 ms. The *N*-glycans were manually identified based on their molecular mass and PGC-LC retention time as well as CID-MS/MS fragmentation pattern of several key glycan structures as detailed above. RawMeat v2.1 (Vast Scientific), GlycoMod (Expasy), Xcalibur Qual Browser v2.2 (Thermo Fisher Scientific) and GlycoWorkBench v2.1 aided the identification process.

### Prediction of MHC-II binding cores and sequon position mapping

All immunopeptide sequences were searched against their specific DR, DQ and DP allotype (MDA-MB-231: DRB1*0701, DRB1*1305, DQA10201-DQB10202, DQA10505-DQB10301, DPA10103-DPB10201, DPA10201-DPB11701 and THP-1: DRB1*0101, DRB1*1501, DQA10101-DQB10501, DQA10102-DQB10602, DPA10103-DPB10201, DPA10202-DPB10402) using NetMHCIIpan 4.1 to predict the MHC-II binding cores of the identified immunopeptides (27). The sequences with the most confident MHC-II binding core predictions from all alleles were compiled. The sequon positions were determined for both the glycosylated immunopeptides and sequon-containing non-glycosylated immunopeptides relative to the binding core. For example, the immunopeptide NSTFVQALVEHVKEE contained the predicted DRB1*0101 binding core FVQALVEHV and displayed an *N*-glycosylation site at position -3 relative to the start of the predicted binding core.

## Results and discussion

Taking a multi-omics system glycobiology approach (**Supplementary Figure S1**), we explored *N*-glycosylation events of endogenous MHC-II-bound immunopeptides by interrogating immunopeptidomics datasets from monocyte-like (THP-1) (18) and triple negative breast cancer-derived (MDA-MB-231) (20) cell lines relative to the cellular *N*-glycoproteome from these biological systems enabled by the availability of matching *N*-glycomics and proteomics data of lysates of THP-1 (19) and MDA-MB-231 (20, 28) cells.

Given the recognised analytical challenges associated with the large-scale identification of glycosylated peptides (29, 30), let alone glycosylated immunopeptides (12), we used a glycomics-guided approach to define a bespoke glycan search space to accelerate the data analysis search and lower the mis-identification rate of glycosylated immunopeptides (25, 31). To this end, we firstly mined the available PGC-LC-MS/MS-based glycomics data of the cellular *N*-glycome of MDA-MB-231 and THP-1 from which 29 and 41 *N*-glycan structures spanning 22 and 29 monosaccharide compositions, respectively, were confidently identified (**Supplementary Tables S1**, **S2**). Hyper-truncated chitobiose core *N*-glycans (GlcNAc_1-2_Fuc_0-1_) play recognised roles in innate immunity and cancer (32, 33) and have been reported on MHC-II immunopeptides (9) yet are ineffectively profiled by PGC-LC-MS/MS-based glycomics; thus, these short *N*-glycans were manually added to the glycan search space.

Utilising the glycomics-guided approach to search the available immunopeptidomics data for *N*-glycosylated immunopeptides, we identified a total of 665 and 353 *N*-glycoPSMs across 145 and 73 unique *N*-glycopeptides mapping to 63 and 31 glycoproteins from MDA-MB-231 and THP-1, respectively (**Supplementary Tables S3**, **S4**). Given that glycopeptide enrichment was not applied to the investigated immunopeptide samples, non-glycosylated immunopeptides were, as expected, more prevalent; in total, 49,806 and 12,109 non-glycosylated PSMs belonging to 10,297 and 4,502 unique non-glycosylated peptides that mapped to 1,768 and 1,129 proteins were detected from the investigated MDA-MB-231 and THP-1 datasets, respectively (**Supplementary Tables S5**, **S6**). Thus, the estimated *N*-glycosylation rate in the investigated MHC-II immunopeptidomics datasets was 1.3% (MDA-MB-231) and 2.8% (THP-1). Another method to estimate the global level of glycosylation in LC-MS/MS datasets is to quantify the presence of a diagnostic HexNAc fragment ion (*m/z* 204.0867) in HCD-MS/MS data (30). As expected, this method produced higher estimates of the *N*-glycosylation level i.e. 9.4% (MDA-MB-231) and 4.5% (THP-1) (**Supplementary Table S7**, **Supplementary Figure S2**), indicating that only a relatively minor fraction of the glycopeptide MS/MS spectra was identified using our search strategy and that the actual rate of *N*-glycosylation may be higher than the 1-3% levels estimated in the two investigated systems.

**Figure 1:**
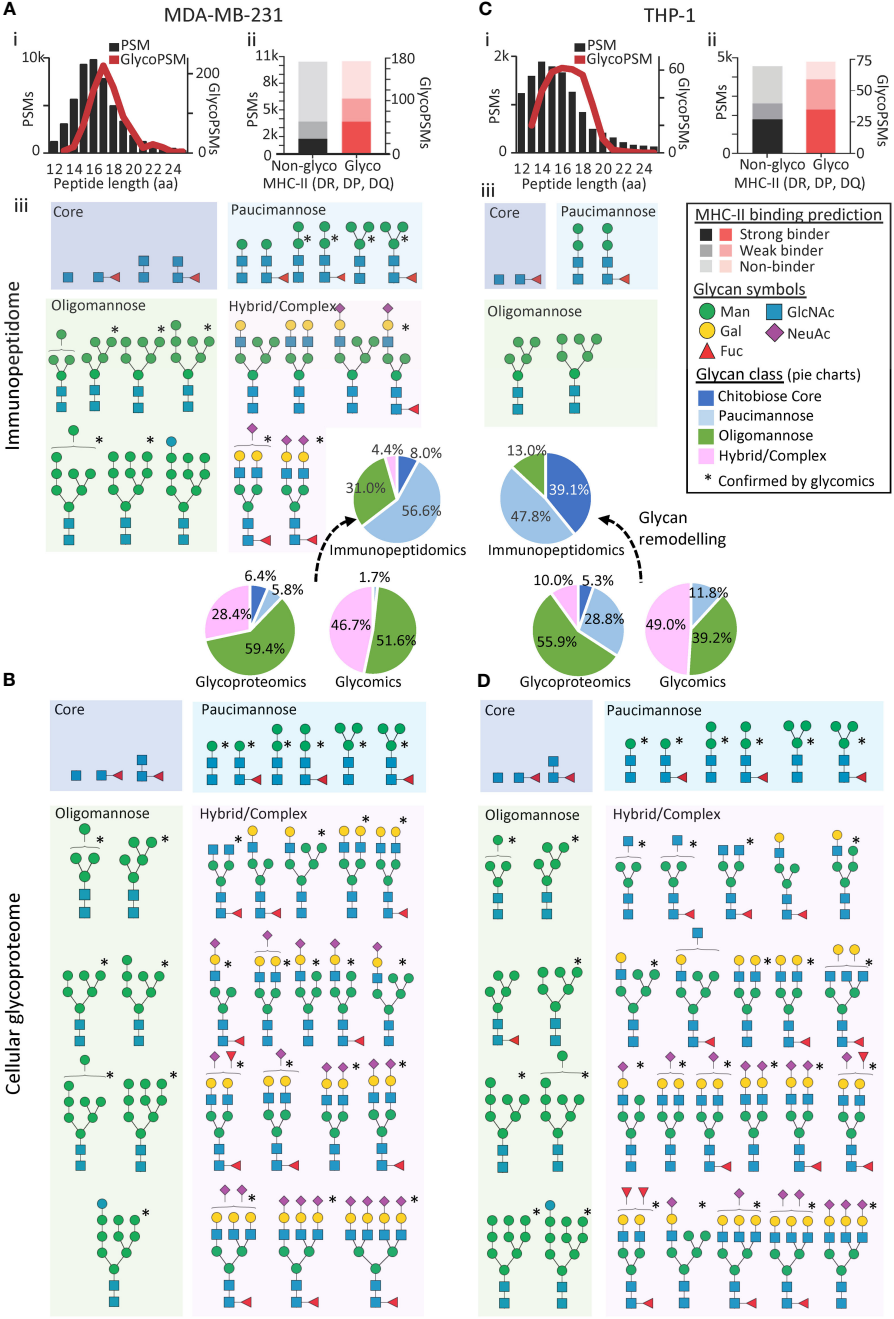
The *N*-glycosylation of MHC-II-bound immunopeptides differs from the cellular glycoproteome in MDA-MB-231 and THP-1. **(A)** MDA-MB-231 immunopeptide length distribution (i), allotype binding specificity (ii) and *N*-glycans (isomer-condensed) identified in available MHC-II immunopeptidomics data (iii) (20). **(B)** MDA-MB-231 cellular *N*-glycans identified using available proteomics data (20). *Structures confirmed from either newly generated *N*-glycomics data of MHC-II immunopeptides isolated from MDA-MB-231 **(A)** or from available MDA-MB-231 *N*-glycomics data (lysate, B) (28). **(C)** THP-1 immunopeptide length distribution (i) allotype binding specificity (ii) and *N*-glycans identified in available MHC-II immunopeptidomics data (iii) (18). **(D)** THP-1 cellular *N*-glycans identified using available proteomics data (19). *Structures confirmed with available THP-1 *N*-glycomics data (lysate) (19). See **Supplementary Figure S5** for a quantitative distribution of all glycan compositions identified by the glycopeptide profiling. See insert for key to the binding prediction, *N*-glycan classes and *N*-glycan structures. *N*-glycans were drawn using the symbol nomenclature for glycomics (36).

Adding confidence in the identified MDA-MB-231 immunopeptides, both the glycosylated and non-glycosylated immunopeptides were found to conform to the expected MHC-II peptide length profile (34, 35) with most identified immunopeptides being between 15-17 amino acid residues (**Figure 1A**i). Strengthening further the confidence in the reported immunopeptides, a considerable proportion of the glycosylated and non-glycosylated MDA-MB-231 immunopeptides were deemed likely MHC-II binders using allotype-specific (HLA-DR, DP, DQ) HLA-binding prediction software (37) (**Figure 1A**ii). Moreover, most of the glycosylated and non-glycosylated MDA-MB-231 immunopeptides showed the expected non-tryptic cleavage pattern (**Supplementary Figure S3A**). Finally, the MDA-MB-231 immunopeptides were found to carry biosynthetically-related glycoforms and formed nested sets, a typical feature of MHC-II immunopeptides (16), generating an additional level of confidence in our findings (**Supplementary Figure S4A**). In total, 23 *N*-glycan structures (compositions) were found to decorate the MDA-MB-231 immunopeptides spanning four distinct glycan classes including chitobiose core (GlcNAc_1-2_Fuc_0-1_), paucimannose (Man_1-3_GlcNAc_2_Fuc_0-1_), oligomannose/oligomannose-like (Glc_0-1_Man_4-9_GlcNAc_2_) and hybrid/complex-type *N*-glycans (**Figure 1A**iii).

Next, we orthogonally confirmed and further detailed the immunopeptide glycosylation features, by generating new PGC-LC-MS/MS-based *N*-glycomics data of isolated MDA-MB-231 immunopeptides. Despite considerable challenges owing to low sample amounts, the glycomics analyses of the immunopeptides confirmed the presence of 12 of the 23 *N*-glycan structures already observed in the MDA-MB-231 immunopeptidome (**Supplementary Table S8**, **Figure 1**, see asterisks). Due to limited sample amounts, our glycomics analysis did not allow for quantitation of the *N*-glycans decorating the isolated MDA-MB-231 immunopeptides, but we observed a qualitative fit to the proteomics-based glycoprofile of the MDA-MB-231 immunopeptidome. Our data demonstrate, for the first time, that PGC-LC-MS/MS-based glycomics of isolated immunopeptides indeed is feasible opening exciting avenues to obtain structural insight into the glycosylation of peptide antigens.

In stark contrast, a similar glycomics-guided search for intact tryptic *N*-glycopeptides in the proteomics data of the cellular fraction (lysate) of MDA-MB-231 revealed with 2,367 *N*-glycoPSMs across 714 unique *N*-glycopeptides mapping to 207 glycoproteins, a cellular glycoproteome rich in oligomannosidic- and complex-type *N*-glycans (**Figure 1B**, **Supplementary Table S9**). Importantly, the dominant oligomannosylation and complex-type *N*-glycosylation of the MDA-MB-231 lysate was confirmed by quantitative glycomics, which also validated most of the *N*-glycan structures observed in the proteomics data (i.e. 29 of 33 *N*-glycans, **Supplementary Table S1**). Given the abundance of oligomannosylation in this data, we cannot rule out that a fraction of the glycoproteins profiled in the lysate was misfolded in the protein quality control or awaiting processing in the secretory machinery; however, the relatively low level (i.e. 4.3% by glycomics, 1.7% by glycopeptide analysis) of an immature *N*-glycan precursor (Glc_1_Man_9_GlcNAc_2_) indicates that this may account for a minor fraction. Despite arising from the same cell lines grown under similar conditions, another limitation of this study is that the multiple -omics datasets were not obtained from the same batch of cultured cells. While some of the observed differences in glycosylation between the immunopeptides and cellular fraction therefore potentially may be attributed to differences in culture conditions and/or passage number, we estimate that such factors account for a relative minor proportion of the prominent differences in glycophenotype of the immunopeptidome and the cellular proteome, aspects that should be systematically explored and further expanded on in the future using cell lines as well as primary cells and tissues.

Reassuringly, both the glycosylated and non-glycosylated immunopeptides identified from the THP-1 cells also conformed to the expected MHC-II peptide profile (**Figure 1C**i). Furthermore, most immunopeptides were predicted MHC-II binders (**Figure 1C**ii), displayed the expected non-tryptic cleavage pattern (**Supplementary Figure S3B**) and contained nested sets (**Supplementary Figure S4B**). With only five *N*-glycan compositions observed, the *N*-glycosylation of the THP-1 immunopeptides appeared relatively homogenous comprising only under-processed (oligomannose, Man_5-6_GlcNAc_2_) and hyper-processed (paucimannose, Man_2_GlcNAc_2_Fuc_0-1_ and chitobiose core, GlcNAc_2_Fuc_0-1_) *N*-glycans (**Figure 1C**iii). Thus, the *N*-glycans observed in the MHC-II immunopeptidome of both THP-1 and MDA-MB-231 were largely in line with the few previous studies that have reported on the *N*-glycosylation of MHC-II-bound immunopeptides (11, 12, 16). Importantly, our findings are also in agreement with the allele-specific findings by Nesvizhskii and colleagues as our predicted MHC-II binders for the DRB1*07:01 and DRB1*01:01 allotypes from MDA-MB-231 and THP-1, respectively, showed similar *N*-glycan type distribution as data retrieved from the HLA-Glyco database (**Supplementary Figure S5**).

Dramatically varying from the simple glycophenotype of the THP-1 immunopeptidome, analysis of the THP-1 lysate revealed with 281 *N*-glycoPSMs across 132 unique *N*-glycopeptides mapping to 44 glycoproteins, a heterogeneous cellular *N*-glycoproteome rich in oligomannosidic- and complex-type *N*-glycans (**Figure 1D**, **Supplementary Table S10**). Importantly, the prevalence of oligomannosylation and complex-type *N*-glycosylation of the THP-1 lysate (the latter being totally absent in the immunopeptidome) was confirmed by quantitative glycomics, which also validated most of the *N*-glycan structures observed in the proteomics data (i.e. 32 of 38 *N*-glycans, **Supplementary Table S2**). Adding yet another level of confidence in the notably different glycophenotypes of the immunopeptidome and the cell lysate across the two investigated systems, structurally related (thus biosynthetically-linked) *N*-glycans were, as expected, identified within both the immunopeptidome and lysates of MDA-MB-231 and THP-1, see **Supplementary Figure S6** for the quantitative distribution of glycans across the two biological systems.

The finding of *N*-glycosylation events of the MHC-II immunopeptidome from MDA-MB-231 and THP-1 prompted us to explore if any patterns or preferences in sequon position within the immunopeptides exist relative to the MHC-II binding core region (**Supplementary Figure S7**). For this purpose, we interrogated both the *N*-glycosylated and non-glycosylated (but sequon-containing, NxS/T, x ≠ P) immunopeptides. Interestingly, the analysis of the glycosylated immunopeptides revealed a preference for sequons flanking both sides of the MHC binding core with the three residues immediately outside the predicted core region being enriched (**Supplementary Figures S7A, C**), a finding supported by the recent work of Bedran et al. (12). These observations indicate that an *N*-glycan installed within the binding core may impede MHC-II immunopeptide processing, loading and/or presentation. However, further studies are required to confirm such speculations and detail how *N*-glycans potentially may hinder, in a position-specific manner, MHC-II presentation processes. As a comparison, we also determined the sequon position of non-glycosylated immunopeptides that contained an unoccupied *N*-linked sequon. Within these non-glycosylated sequon-containing immunopeptides, sequons appeared more prevalent within the predicted binding core (**Supplementary Figures S7B, D**).

We also searched the THP-1 and MDA-MB-231 immunopeptidomics datasets for de-*N*-glycosylation events to assess the prevalence of formerly *N*-glycosylated MHC-II immunopeptides relative to the spontaneous Asn deamidation rate as determined using immunopeptides not carrying a sequon for *N*-glycosylation (**Supplementary Table S11**). Since the de-*N*-glycosylation rate (MDA-MB-231: 3.1%; THP-1: 5.1%) appeared similar to and only mildly above the spontaneous Asn deamidation rate (MDA-MB-231: 1.3%; THP-1: 3.2%), this analysis indicates that de-*N*-glycosylation is an infrequent event of MHC-II-bound immunopeptides within the two investigated biological systems. The insignificant de-*N*-glycosylation of the MHC-II-bound immunopeptides is in agreement with our previous work that suggests that de-*N*-glycosylation events by the cytosolic de-*N*-glycosylation enzymes instead is more common for the MHC-I-bound immunopeptides known to traffic the cytosol during the peptide antigen presentation process (38).

To document further the glycan remodelling of the MHC-II immunopeptidome, we next profiled the glycosylation of specific cellular glycoproteins and contrasted their glycoprofiles to the glycosylation of the MHC-II-bound immunopeptides arising from the same proteins. To enable a comprehensive analysis, we selected three cell surface glycoproteins i.e. integrin alpha-2, transferrin receptor-1 and protocadherin FAT1 all detected in MDA-MB-231 cells and two lysosomal glycoproteins i.e. lysosome associated membrane protein 1 (LAMP-1) and prosaposin detected in MDA-MB-231 and THP-1 cells, respectively.

**Figure 2:**
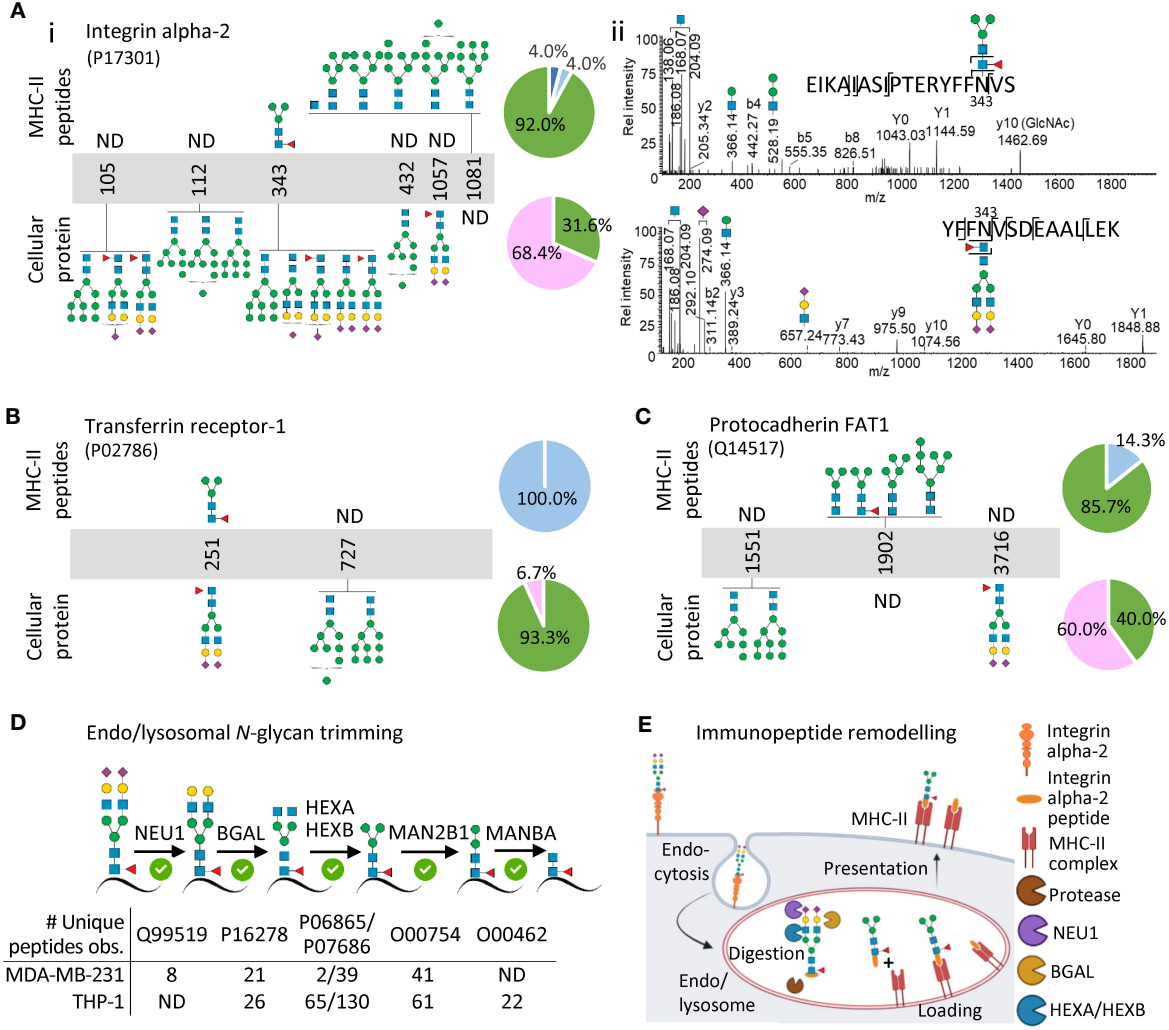
Protein- and site-specific glycoprofiling indicate that extensive glycan remodelling accompanies MHC-II presentation. **(A)** Site-specific *N*-glycoprofiling of immunopeptides from integrin alpha-2 (top) and of tryptic peptides from cellular integrin alpha-2 (bottom) (i). The accompanying pie charts plot the distribution of observed *N*-glycans for integrin alpha-2 (across all observed sites) within the immunopeptidomics and cellular proteomics data. Representative HCD-MS/MS spectra documenting the remodelling of a prominent *N*-glycan of an immunopeptide spanning the N343 site of integrin alpha-2 (top) relative to a prominent *N*-glycan from the same site of cellular integrin alpha-2 (bottom) (ii). Similar glycoprofiling was performed for **(B)** transferrin receptor-1 and **(C)** protocadherin FAT1 (see **Supplementary Figure S4** for spectral evidence). MDA-MB-231 data was used for **(A–C)**. **(D)** Identification of key glycoside hydrolases (putatively responsible for the observed glycan remodelling of MHC-II immunopeptides) in the MDA-MB-231 and THP-1 cellular proteomics datasets. **(E)** Model schematically illustrating how glycan remodelling may accompany MHC-II immunopeptide presentation. Cell surface glycoproteins carrying complex-type *N*-glycans (here integrin alpha-2) are endocytosed and undergo extensive endo/lysosomal trimming of the polypeptide chains and the conjugated glycans to produce immunopeptides carrying remodelled *N*-glycans displayed by MHC-II molecules on the cell surface. See **Figure 1** for key to glycan classes used in pie charts and glycan cartoons.

Integrin alpha-2 is a key adhesin on the cell surface of cancer cells and other tissues (39). In line with a recent study that site-specifically profiled the *N*-glycans of integrin alpha-2 from human ovarian cancer cell lines (40), our cellular proteomics data revealed that this glycoprotein carries complex/hybrid-type (68.4%) and oligomannose-type (31.6%) *N*-glycans across the five profiled sites (**Figure 2A**i). In sharp contrast, the MHC-II-bound immunopeptides originating from integrin alpha-2 were devoid of complex/hybrid-type *N*-glycans and instead overwhelmingly carried oligomannose (92.0%) and low levels of paucimannose (4.0%) and chitobiose core (4.0%). At the site-specific level, dramatic remodelling was demonstrated for Asn343 of integrin alpha-2 that was covered by both the proteomics and immunopeptidomics data; this comparative analysis clearly illustrated the transformation of an elongated biantennary sialo-*N*-glycan on a tryptic peptide to a paucimannosidic *N*-glycan on an MHC-II-bound immunopeptide (**Figure 2A**ii). Similar glycan remodelling was demonstrated for transferrin receptor-1 and protocadherin FAT1, two other important cell surface glycoproteins, documenting further the (near-)absence of complex-type *N*-glycosylation on the MHC-II-bound immunopeptides (**Figures 2B, C**, **Supplementary Figure S8**). These protein-centric findings of glycan remodelling are in line with and expand on similar observations made by Nesvizhskii and colleagues for the cell surface-resident protein prolow-density lipoprotein receptor-related protein 1 (12). To support further the abundance of complex- and oligomannosidic-type *N*-glycosylation of the cell surface proteins, we also used a gene ontology approach to glycoprofile (indirectly) the global cell surface glycoproteome of the MDA-MB-231 cell system (**Supplementary Table S12**, **Supplementary Figure S9**). As expected, this global analysis confirmed a high prevalence of under-processed oligomannosidic-type *N*-glycans (48.3%) and elaborate hybrid/complex-type *N*-glycans (47.8%) on the MDA-MB-231 cell surface and thus supports that glycan remodelling accompanies MHC-II immunopeptide presentation.

The analysis of two lysosomal glycoproteins i.e. LAMP-1 and prosaposin showed comparatively less glycan remodelling associated with MHC-II presentation (**Supplementary Figure S10**). Notably, the soluble prosaposin residing in the hydrolytic environment of the THP-1 lysosomes already exhibited a truncated glycophenotype rich in paucimannosidic- and chitobiose core-type *N*-glycans in the cellular (lysate) fraction mimicking the heavily processed *N*-glycans decorating the MHC-II-bound immunopeptides of prosaposin. LAMP-1 carried both complex- and paucimannosidic-type *N*-glycans likely due to its presence on both the cell surface and within the lysosome (41–43). In line with previous observations (11), these findings suggest that prolonged exposure to an array of lysosomal glycosidases facilitates a truncated glycophenotype of endogenous lysosomal glycoproteins, which are consequently subjected to less glycan remodelling upon MHC-II immunopeptide presentation relative to the complex *N*-glycan-rich cell surface proteins that are not exposed to glycoside hydrolases until entering the MHC-II processing pathway in the endo/lysosomal system (thus undergoing relatively more remodelling), aspects we are currently exploring in greater detail.

To further support that glycan remodelling accompanies MHC-II immunopeptide presentation, we interrogated the cellular proteomics data for glycoside hydrolases previously implicated in *N*-glycan trimming within the *N*-glycoprotein truncation pathway (44, 45). Excitingly, most of the lysosomal glycoside hydrolases expected to be responsible for the trimming of elongated sialo-*N*-glycans to short paucimannosidic- and chitobiose core-type *N*-glycans were confidently identified in both MDA-MB-231 and THP-1 (e.g. BGAL, HEXA, HEXB, MAN2B1) (**Figure 2D**). While these observations support our glycan remodelling hypothesis, additional studies are required to confirm and pinpoint the exact involvement of individual glycoside hydrolases in the *N*-glycan trimming process. Beyond the speculation that the solvent-inaccessible oligomannosidic *N*-glycans on the surface of glycoproteins may confer steric protection from the action of lysosomal α-mannosidases (46), the peculiar co-occurrence of under-processed oligomannosidic structures on the immunopeptides alongside the hyper-processed *N*-glycans remains mechanistically unexplained.

By overlaying our findings of remodelled immunopeptide *N*-glycans observed across two different biological systems on the otherwise well-established MHC-II processing and presentation pathway, we propose that the dramatic glycophenotypic transformation of endogenous cell surface glycoproteins occur after their endocytosis when the cell surface glycoproteins are exposed to the hydrolytic endo/lysosomal environment (**Figure 2E**). As such, our model suggests that complex-/hybrid-type *N-*glycoproteins rather than relatively unprocessed oligomannosidic *N*-glycoproteins predominantly are trimmed during MHC-II processing as supported by a high prevalence of core fucosylation of the truncated *N*-glycans identified on the MHC-II-bound immunopeptides (11, 12). Core fucosylation is namely known to be transferred selectively to complex-/hybrid-type *N*-glycoprotein substrates early in the biosynthetic pathway and is therefore a reliable indicator that the truncated glycans predominantly arise from pruning of larger complex-/hybrid-type *N*-glycans through the *N-*glycoprotein truncation pathway (44, 45). However, important spatiotemporal details such as the chronological order of the glycan- and polypeptide-based processing relative to the MHC-II peptide antigen loading and presentation processes remain unknown.

## Conclusion

We have here applied a multi-omics approach to enable a comprehensive view of the *N-*glycosylation of endogenous MHC-II-bound immunopeptides relative to the cellular *N*-glycoproteome from cultured monocyte-like and breast cancer-derived cell lines. We find evidence to support the notion that both under-processed and extensively remodelled *N*-glycans prevalently modify MHC-II immunopeptides arising from endogenous glycoproteins suggesting that our immune system has evolved previously unknown mechanisms to dynamically shape and fine-tune the immune response through diverse repertoires of hitherto largely ignored molecular patterns. Notably, the glycan remodelling observed in both investigated biological systems is of potentially functional relevance as it produces an abundance of exposed α-mannosylated glycoepitopes on the MHC-II-bound immunopeptides in the form of oligo- and paucimannosidic-type *N*-glycans that are otherwise predominantly hidden from the extracellular environment under homeostatic conditions (47). Our study, which suggests that glycan remodelling accompanies MHC-II immunopeptide presentation and a recent study by other investigators, which demonstrates the wide-spread occurrence of *N*-glycosylation on the MHC-II immunopeptidome (12) collectively raise enticing questions about the role(s) glycosylation may play on immunopeptide processing, loading, presentation and T-cell receptor recognition in the context of immune surveillance.

## Data availability statement

Publicly available datasets were analysed in this study. This data can be found here: PXD015646 (Pride) PXD005169 (Pride) PXD023044 (Pride) PXD023038 (Pride) MSV000083727 (Massive).

## Ethics statement

Ethical approval was not required for the studies on humans in accordance with the local legislation and institutional requirements because only commercially available established cell lines were used.

## Author contributions

HG: Conceptualisation, Investigation, Methodology, Visualisation, Writing – original draft, Writing – review & editing, Data curation, Formal Analysis, Validation. RK: Conceptualisation, Data curation, Funding acquisition, Investigation, Methodology, Resources, Supervision, Validation, Visualisation, Writing – review & editing. SC: Resources, Validation, Writing – review & editing. GG: Methodology, Resources, Writing – review & editing. JF: Software, Validation, Writing – review & editing. AP: Funding acquisition, Resources, Validation, Writing – review & editing. NC: Conceptualisation, Data curation, Investigation, Methodology, Resources, Software, Supervision, Validation, Visualisation, Writing – review & editing. MT-A: Conceptualisation, Funding acquisition, Investigation, Methodology, Project administration, Resources, Supervision, Visualisation, Writing – original draft, Writing – review & editing, Validation.
