# Supplementary material for: Profound N-glycan remodelling accompanies MHC-II immunopeptide presentation
Source: Front Immunol. 2023 Nov 8;14:1258518. doi: 10.3389/fimmu.2023.1258518 (PMC10663315; doi:10.3389/fimmu.2023.1258518)
Supplement: Supplementary file 1 [file DataSheet_1.pdf]

Supporting Information (SI)

for

Profound *N*-glycan remodelling accompanies MHC-II immunopeptide presentation

Hayley Goodson[1], Rebeca Kawahara[1,2], Sayantani Chatterjee[1,3], Gabriel Goncalves[4], Joshua Fehring[4], Anthony W. Purcell[4], Nathan P. Croft[4]*, and Morten Thaysen-Andersen[1,2]*

[1]School of Natural Sciences, Macquarie University, Sydney NSW 2109, Australia

[2]Institute for Glyco-core Research (iGCORE), Nagoya University, Nagoya, Japan

[3]Department of Biochemistry & Cell Biology, Boston University Chobanian & Avedisian School of Medicine, Boston, Massachusetts, USA

[4]Department of Biochemistry and Molecular Biology, Monash Biomedicine Discovery Institute, Monash University, Clayton, VIC 3800, Australia

Running title: MHC-II immunopeptides carry remodelled *N*-glycans

Key words: MHC-II, immunopeptides, *N*-glycans, glycan remodelling, glycomics, glycoproteomics

* Joint corresponding authors

Associate Prof Morten Thaysen-Andersen, PhD
Office 333, Building 4WW
School of Natural Sciences
Macquarie University
NSW-2109 Macquarie Park
Sydney, Australia.
E-mail: morten.andersen@mq.edu.au
Phone: +61 2 9850 7487

Dr Nathan P. Croft, PhD
Room 219, 15 Innovation Walk
Biomedicine Discovery Institute
Monash University
VIC-3800, Clayton
Melbourne, Australia
Email: nathan.croft@monash.edu
Phone: +61 3 9902 0473

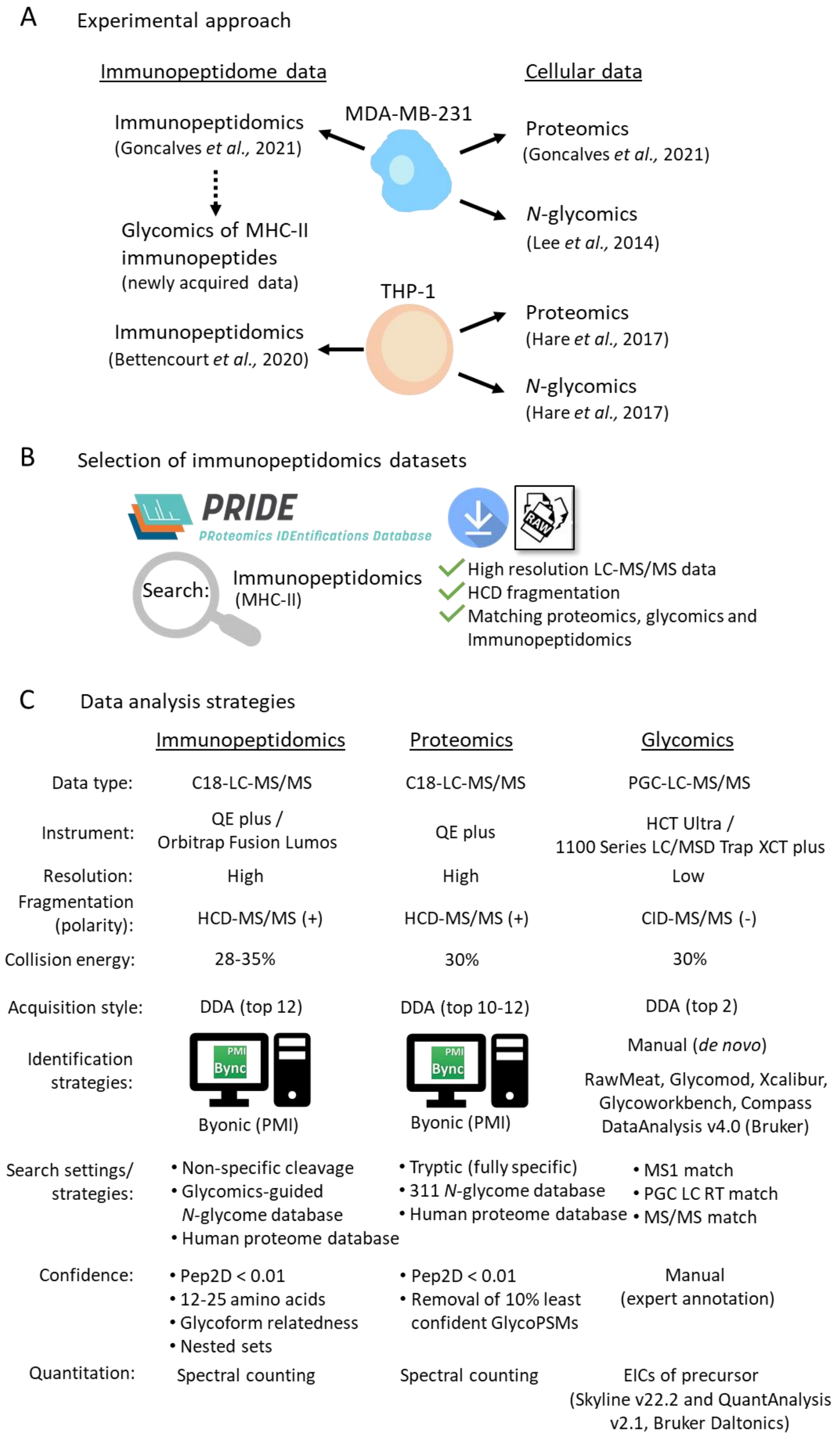

Supplementary Figure S1. **Study overview**. **A**) Overview of the multi-omics datasets of the investigated MDA-MB-231 and THP-1 cell lines. **B**) Search parameters employed to identify MCH-II immunopeptidomics datasets suitable for this study. **C**) Key search strategies and data analysis parameters used to analyse the immunopeptidomics (left), proteomics (middle) and glycomics (right) datasets.

A

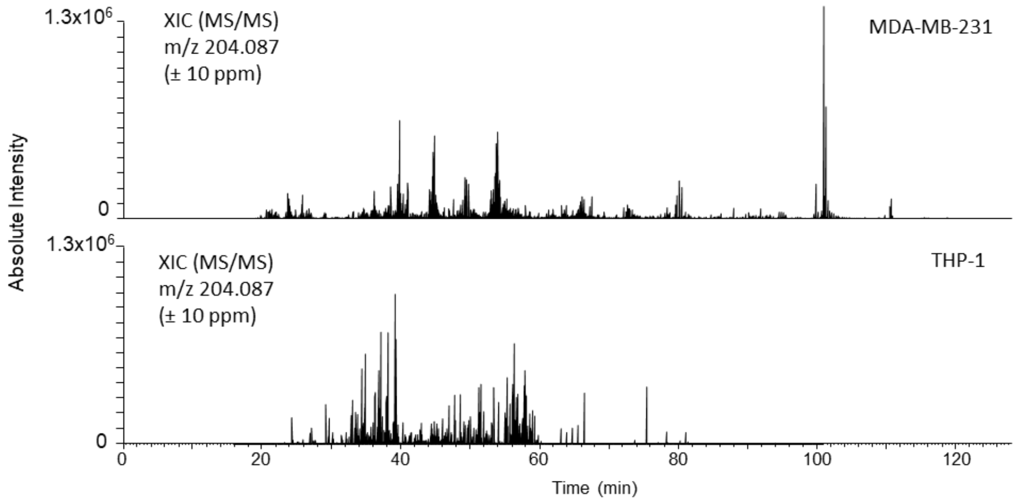

B

	MDA-MB-231	THP-1
Single LC-MS/MS run (Panel A)		
Total MS/MS spectra:	37,466	24,384
Glycopeptide MS/MS spectra (proportion of all):	6,255 (16.7%)	1,466 (6.0%)
All LC-MS/MS runs		
Total MS/MS spectra:	982,005	287,271
Glycopeptide MS/MS spectra (proportion of all):	92,119 (9.4%)	12,806 (4.5%)

Supplementary Figure S2. **Glycopeptide prevalence in MDA-MB-231 and THP-1 immunopeptidomics datasets. A**) XIC (MS/MS) traces of *m/z* 204.087 ± 10 ppm fragment ions diagnostic for the presence of glycosylation in HCD-MS/MS spectra within a representative immunopeptidomics LC-MS/MS file from the MDA-MB-231 (top) and THP-1 (bottom) cell lines. **B**) Counts of all MS/MS spectra and the glycosylated MS/MS spectra in both a single LC-MS/MS file (as depicted in Panel A) and across all available LC-MS/MS files. See **Supplementary Table S7** for details.

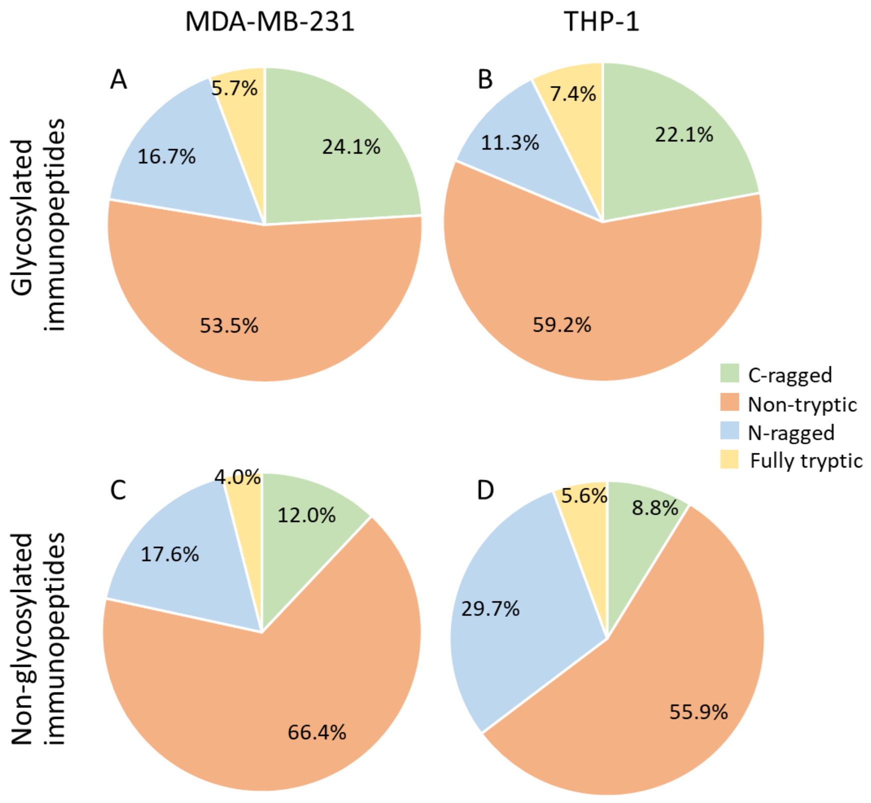

Supplementary Figure S3. Cleavage pattern of the identified immunopeptides. Proportion of immunopeptide (glyco)PSMs that were either fully tryptic, semi-tryptic (N- or C-ragged) or non-tryptic assessed separately for the identified glycosylated immunopeptides from **A)** MDA-MB-231 and **B)** THP-1 and the identified non-glycosylated immunopeptides from **C)** MDA-MB-231 and **D)** THP-1. See insert for key.

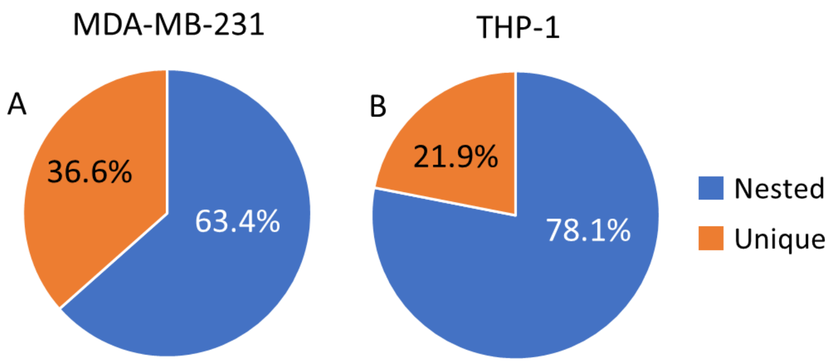

Supplementary Figure S4. **Nested immunopeptide sequences**. Proportion of identified glycosylated immunopeptides that formed nested sets from **A**) MDA-MB-231 and **B**) THP-1.

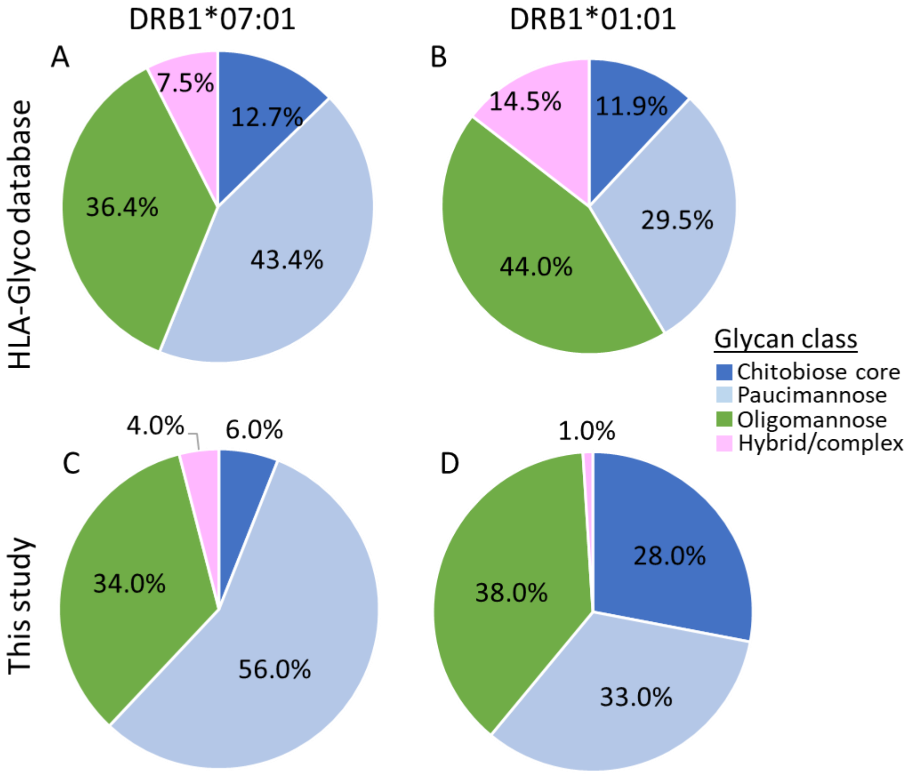

E

Allele type	Number of reported glycosylated immunopeptides	
	HLA-Glyco database	This study
DRB1*07:01	788	50
DRB1*01:01	847	100

Supplementary Figure S5. **Comparison of the glycosylated immunopeptides from the HLA-Glyco database and our reported glycosylated immunopeptides**. Glycan class distribution of the reported glycosylated immunopeptides from the HLA-glyco database [12] predicted to bind the HLA-II alleles **A)** DRB1*07:01 and **B)** DRB1*01:01. Glycan class distribution of our reported glycosylated immunopeptides predicted to bind the HLA-II alleles **C)** DRB1*07:01 from MDA-MB-231 and **D)** DRB1*01:01 from THP-1. **E)** Tabulated summary of the number of reported glycosylated immunopeptides from both studies.

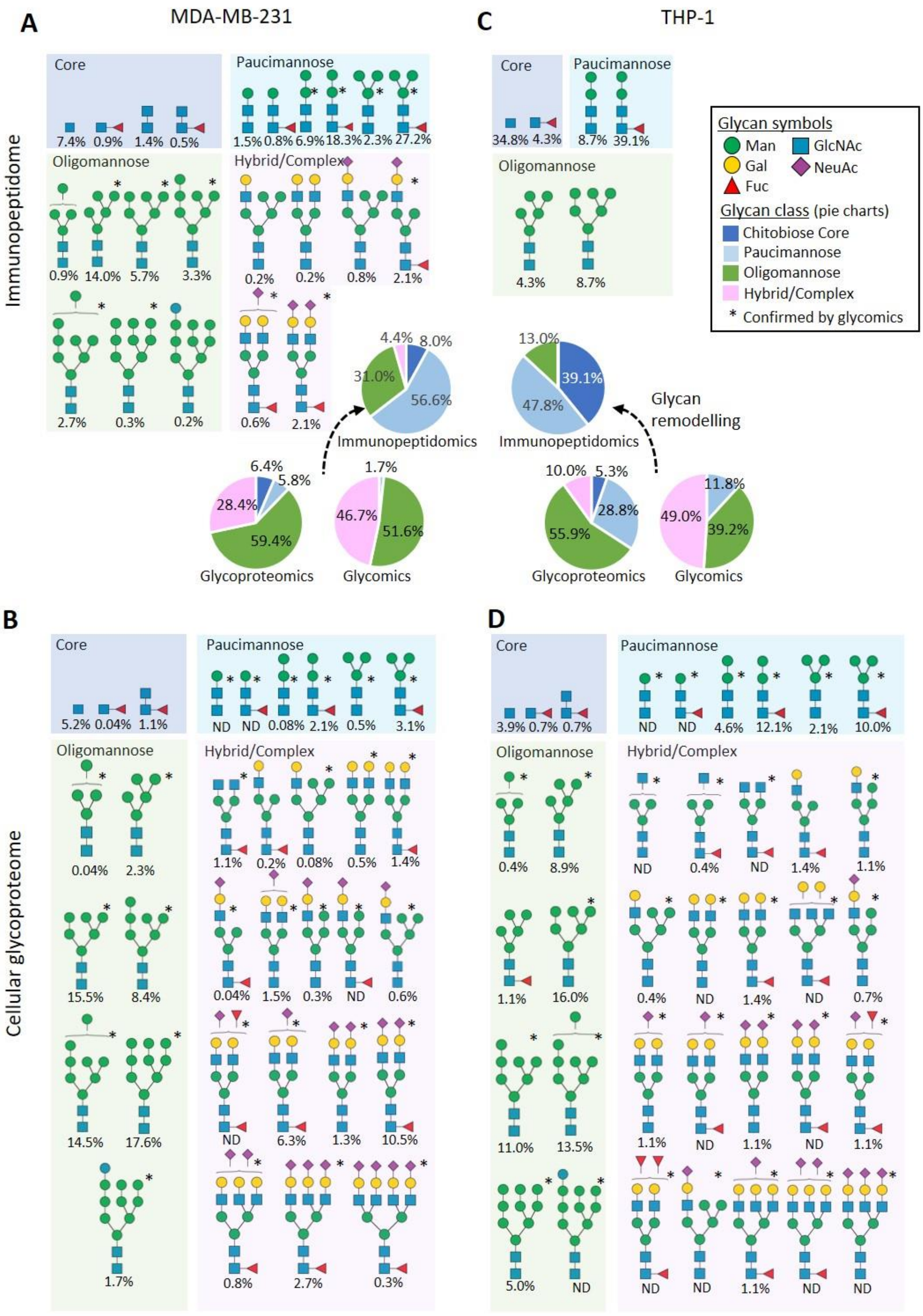

Supplementary Figure S6. Distribution of all *N*-glycan compositions identified by glycopeptide profiling. The relative abundances for each glycan composition as identified by Byonic-based glycopeptide analysis in the MDA-MB-231 **A)** glycomics and **B)** glycoproteomics datasets, as well as in the THP-1 **C)** glycomics and **D)** glycoproteomics datasets. ND, glycoform not detected with glycopeptide analysis.

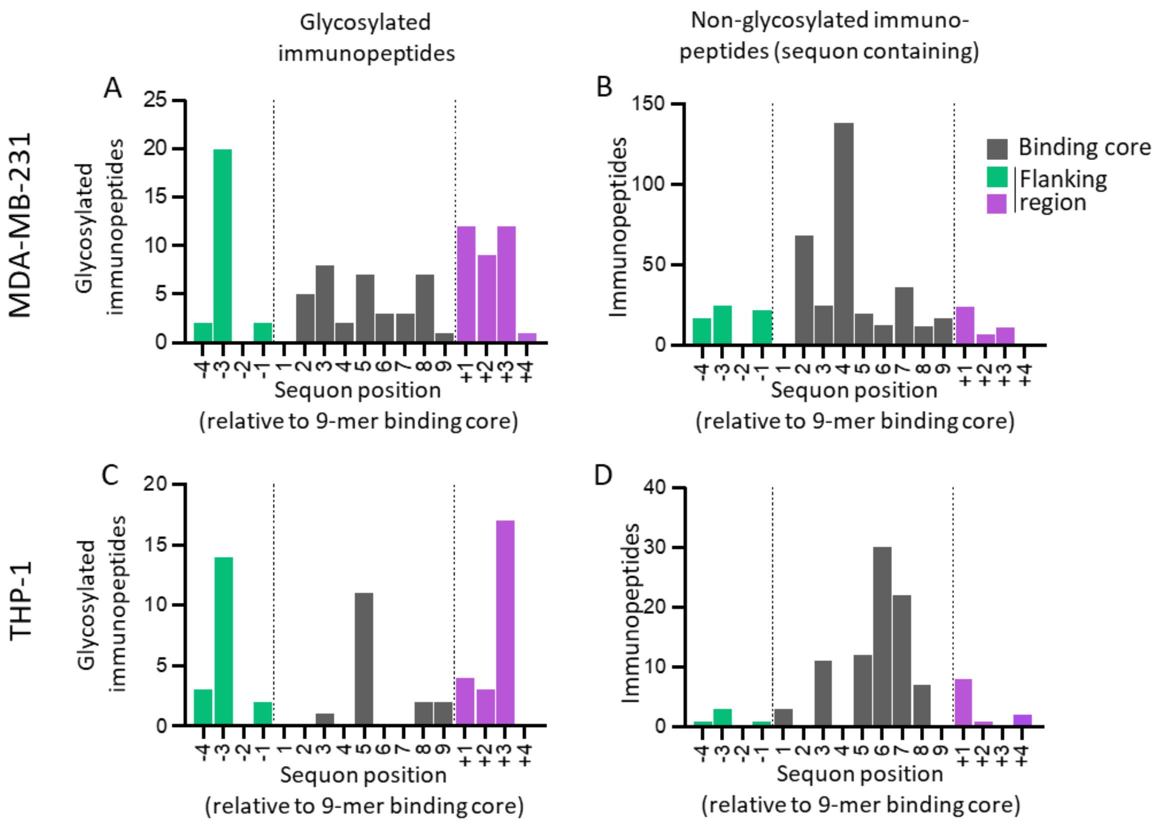

Supplementary Figure S7. **Position of *N*-glycosylation site (sequon) within the identified immunopeptides relative to the MHC-II binding core**. Distribution of the sequon position of **A**) glycosylated and **B**) non-glycosylated (but sequon-containing) MDA-MB-231 immunopeptides relative to the binding core (in grey) as predicted using an MHC-II allotype-specific prediction algorithm. Distribution of the sequon position of **C**) *N*-glycosylated and **D**) non-glycosylated (sequon-containing) THP-1 immunopeptides relative to predicted MHC-II binding cores.

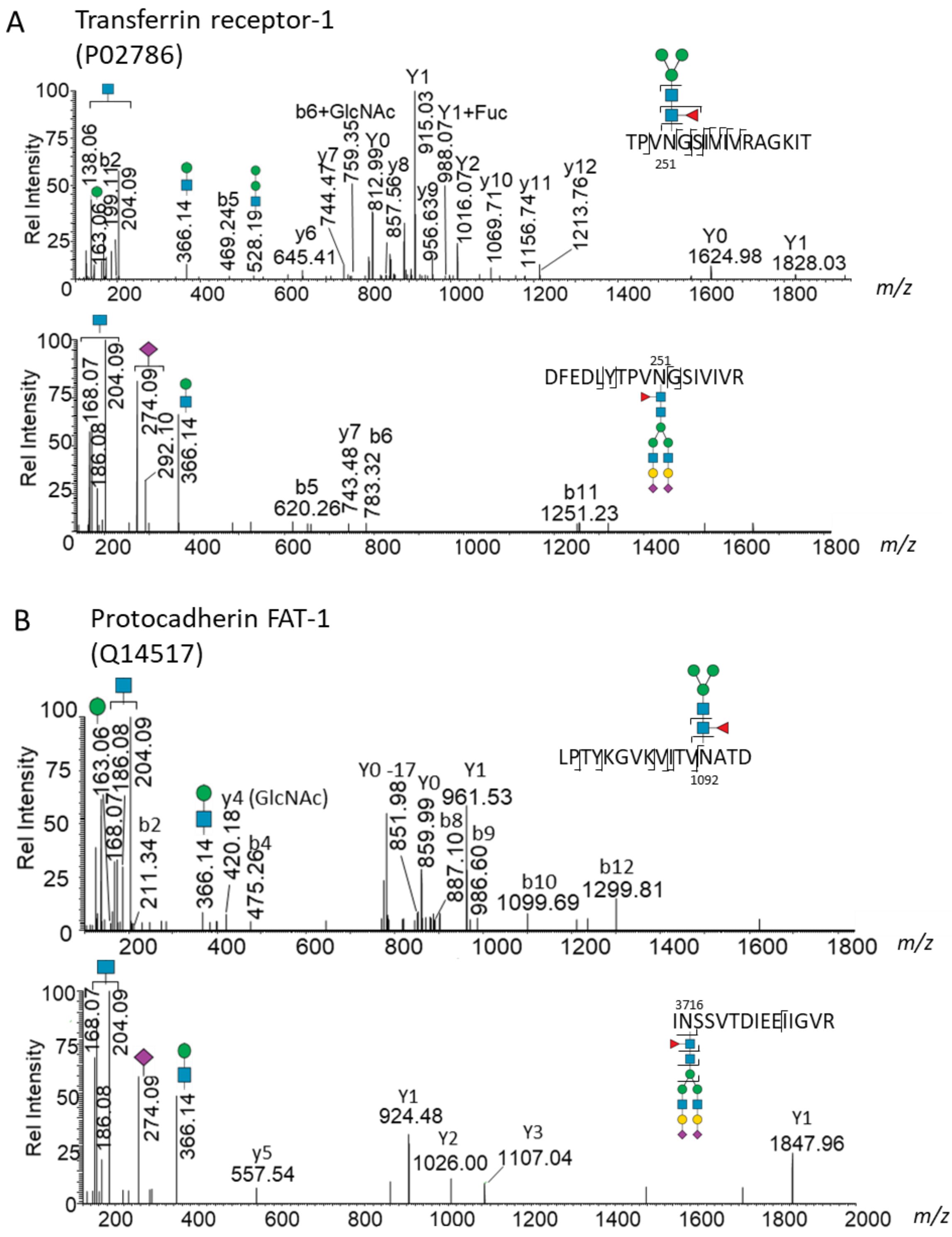

Supplementary Figure S8. **Dramatic glycan remodelling of cell surface proteins upon MHC-II immunopeptide presentation**. Representative HCD-MS/MS spectra of glycosylated MHC-II immunopeptides (top spectra) and tryptic glycopeptides (bottom spectra) belonging to key cell surface glycoproteins including **A**) transferrin receptor 1 and **B**) protocadherin FAT-1 both expressed by the MD-MBA-231 cell line. For protocadherin FAT-1, the same glycosylation site was not covered by both the immunopeptidomics and cellular proteomics data hence different sites are shown. See Figure 1 for key to the *N*-glycan classes and glycan symbols.

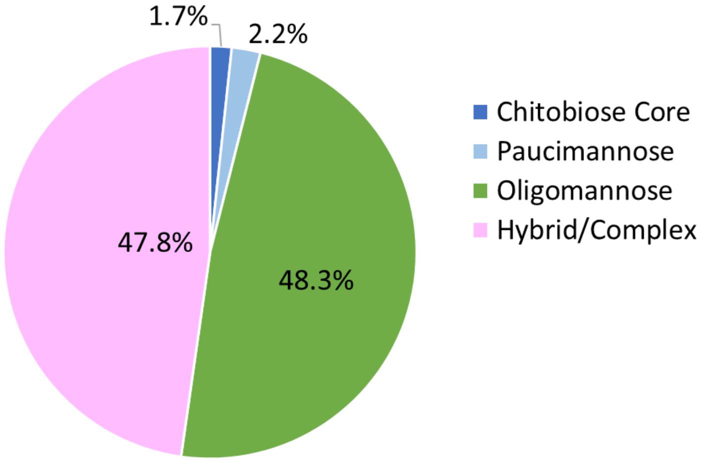

Supplementary Figure S9. Cell surface glycosylation of MDA-MB-231 cells. Glycan type distribution of cell surface/plasma membrane-located glycoproteins from MDA-MB-231 cells identified using a GO annotation approach.

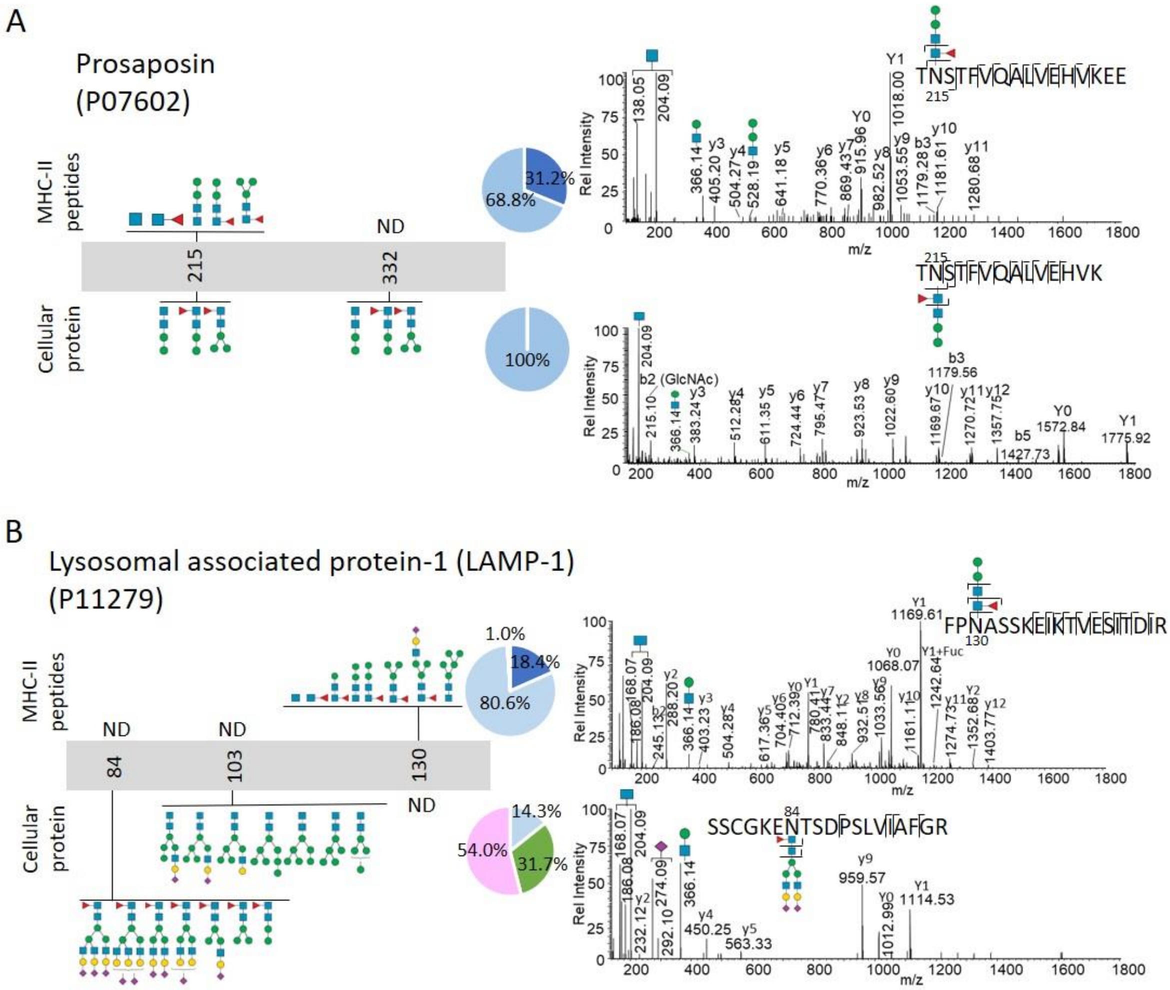

Supplementary Figure S10. **Limited glycan remodelling of lysosomal proteins upon MHC-II immunopeptide presentation**. Overview of the site-specific glycosylation of immunopeptides identified in the MHC-II immunopeptidomics data relative to the glycoprofile of the cellular proteins identified from matching proteomics data of two lysosome-resident glycoproteins including **A**) prosaposin expressed by the THP-1 cell line and **B**) LAMP-1 expressed by the MD-MBA-231 cell line. Representative HCD-MS/MS spectra of a glycosylated MHC-II immunopeptide (top spectra) and a tryptic glycopeptide from the cellular glycoproteins (bottom spectra) are shown. See **Figure 1** for key to the *N*-glycan classes and glycan symbols.
